# The role of hypofractionated radiotherapy for the definitive treatment of localized prostate cancer: early results of a randomized trial

**DOI:** 10.7150/jca.35510

**Published:** 2019-10-16

**Authors:** Petros Alexidis, Dimitris Dragoumis, Sotirios Karatzoglou, Konstantinos Drevelegas, Ioannis Tzitzikas, Konstantinos Hatzimouratidis, Ioannis Chrisogonidis, Dimitris Giannakidis, Charilaos Koulouris, Athanasios Katsaounis, Nikolaos Michalopoulos, Haidong Huang, Qiang Li, Zoi Aidoini, Varbara Fyntanidou, Aikaterini Amaniti, Wolfgang Hohenforst-Schmidt, Elena Maragouli, Savvas Petanidis, Paul Zarogoulidis, Konstantinos Sapalidis, Christoforos Kosmidis, Konstantinos Romanidis, Panagoula Oinkonomou, Anastasios Vagionas, Iason Nikolaos-Katsios, Aris Ioannidis, Konstantina Boniou, Isaak Kesisoglou

**Affiliations:** 1 Department of Radiation Oncology, Interbalkan European Medical Center; Thessaloniki, Greece.; 2 Department of Radiation Oncology, AHEPA University Hospital of Thessaloniki, Faculty of Medicine, School of Health Sciences, Aristotle University of Thessaloniki, Greece.; 3 Department of Urology, Papageorgiou hospital of Thessaloniki, Faculty of Medicine, School of Health Sciences, Aristotle University of Thessaloniki, Greece.; 4 3rd Department of Surgery, AHEPA University Hospital, Aristotle University of Thessaloniki, Medical School, Thessaloniki, Greece.; 5 The Diagnostic and Therapeutic Center of Respiratory Diseases, Shanghai East Hospital, Tongji University, Shanghai, China.; 6 Anesthisiology Department, AHEPA University Hospital, Aristotle University of Thessaloniki, Medical School; Thessaloniki, Greece.; 7 Sana Clinic Group Franken, Department of Cardiology/Pulmonology/Intensive Care/Nephrology, "Hof" Clinics, University of Erlangen, Hof, Germany.; 8 Oncology Department, University of Thessali, Larissa, Greece.; 9 Department of Pulmonology, I.M. Sechenov First Moscow State Medical University; Moscow, 119992, Russian Federation.; 10 Neurosurgical Department, ``G. Papanikolaou`` General Hospital, Thessaloniki, Greece; 11 Radiology Department, Everlight Radiology, U.K; 12 Second Department of Surgery, University Hospital of Alexandroupolis, Medical School, Democritus University of Thrace, 68100 Alexandroupolis, Greece; 13 Oncology Department, General Hospital of Kavala, Greece; 14 Radiology Department, ``Theageneio`` Cancer Hospital, Thessaloniki, Greece

**Keywords:** hypofractionation, early toxicity, prostate cancer

## Abstract

**Background:** Prostate cancer is considered to have a special biology which could affect the radiation therapy result based on the selected fractionation scheme. We present the preliminary results of a randomized trial comparing conventionally and hypofractionated radiation therapy for prostate cancer.

**Methods:** Patients included in the study had localized prostate cancer (cT1c-T3bN0M0) and were randomly assigned to mild hypofractionated (72 Gy in 32 fractions, arm1) or conventionally fractionated (74 Gy in 37 fractions, arm2) radiation therapy treatment with Volumetric Arc Therapy technique. The treatment was delivered only to the prostate with or without the seminal vesicles according to physician's discretion and hormone therapy was optional according to the disease stage and comorbidities. Here we present the preliminary results of acute toxicity from the gastrointestinal (GI) and genitourinary (GU) system.

**Results:** Between 2015 and 2016, 139 patients were enrolled. 67 patients were treated with conventional fractionation and 72 were treated with hypofractionation. Grade≥ 2 toxicity from GU and GI was observed in 23 and 21 patients (31,9% vs 31,3%, p=0,79) and 15 and 12 (20,8% vs 17,9%, p=0,6) for arm1 and arm2 respectively. No statistically significant differences were observed between arms in the incidence of early toxicity. There was no correlation observed between patient characteristics and toxicity from either GU or GI.

**Conclusions:** Hypofractionated radiotherapy appears to be equally tolerated compared to conventional fractionation in the early setting. Longer follow up is needed to assess the late toxicity profile of the patients and any potential differences between the control and experimental arm.

## Introduction

Radiation therapy is one of the most appropriate treatments for localized prostate cancer for all stages (low intermediate and high risk) and is associated with long term disease control. In the US there are 164000 new cases of prostate cancer every year and radiotherapy is among the most commonly used treatments for the disease. There are various studies demonstrating an advantage of dose escalation for disease control 1-6, although an advantage in overall survival is less clear and currently 75, 6 - 81 Gy is considered the standard of care for conventional fractionation 7-9. Modern radiation therapy methods like Volumetric Arc Therapy can achieve a superior conformity of the dose distribution and focus the dose with much higher accuracy to the target, protecting this way the normal tissues 7, 10-13. The use of these techniques has been associated with significantly less toxicity from the surrounding healthy organs, with volumetric modulated arc therapy (VMAT) therapy being now the standard of care for the treatment of prostate cancer 13.

Additionally, there has been a lot of discussion on the fractionation size for the treatment of prostate cancer. Specifically, the association between the total isoeffective dose and its effect on tissues (cancer and normal tissues) is described by the linear quadratic model which uses two constants, α and β. the α/β ratio is determining the sensitivity of the corresponding tissue to the changes in dose per fraction and its value is estimated to be approximately 10 for cancer and 3 for normal tissues. Prostate cancer though is considered to have an α/β ratio of as low as 1.5, lower than that of the normal tissues, which could have important therapeutic implications 14, 15. Delivering the treatment with less fractions and a higher dose per fraction could cause a higher dose escalation effect on prostate compared to normal tissues. This in turn could lead to better disease control, less toxicity and more convenience for the patients.

In this randomized trial we use VMAT technique to compare conventional fractionation of 2Gy/fr to a mild hypofractionated therapy schedule of 2.25 Gy/fr and the effect of these treatments on disease control and toxicity. Here we present the results on acute toxicity during the first 5 months from start of RT.

## Methods

### Patients

We recruited patients between 40 and 85 years old with histologically proven localized prostate cancer (cT1c-cT3bN0M0), a prostate specific antigen (PSA) of ≤40 ng/ml and a WHO performance status of 0-2. Patients were excluded if they had received pelvic irradiation in the past, if they had undergone any type of prostatectomy (suprapubic or transurethral), if they suffered from inflammatory bowel disease, patients with a history of bladder cancer or transurethral resection of bladder tumor (TUR BT) or patients with impaired urinary function. Additionally we excluded patients with a calculated risk of lymph node involvement ≥5% 16, those with T3 disease and GS ≥ 8, T3 disease and PSA>10 ng/ml, GS 8-9 and stage T3 or T4 or PSA >10 ng/ml. Workup included digital examination, PSA measurement before treatment, biopsy with a positive results for cancer, CT of the pelvis and abdomen for all patients, pelvic MRI and bone scan for patients with T3-T4 stage, PSA >20 ng/ml or GS 8-9 or for those with symptoms. This trial was approved by the medical ethics committee of Aristotle University of Thessaloniki. All patients provided written informed consent.

### Procedures

Androgen deprivation therapy was given 2 months before the initiation of radiation therapy and consisted of an LHRH analogue combined with initial anti androgen to reduce testosterone flair. Androgen deprivation therapy (ADT) duration was 6 months for patients with intermediate risk disease and 2-3 years for patients with high risk disease according to the physician's discretion. CT simulation was performed by acquiring a 3 mm slice CT of the pelvis from L4 vertebra up to the ischial tuberosities. The patient was instructed to use an enema the previous day and drink 500 ml of water 45 minutes before CT scan. The prostate with or without the seminal vesicles were then delineated. Decision on, if and to which extend, the seminal vesicles would be treated was based on estimation of risk for seminal vesicles involvement 17 and the patients were categorized into three groups. The first group included patients with risk of SV involvement of less than 10% and only the prostate was treated to the maximum dose. The second group included patients with 10-25% risk of SV involvement and the prostate together with the proximal 1 cm of the SV was treated to the maximum dose while the rest of the SV was treated with a lower dose of 56 Gy in the conventional RT arm and 58.9 Gy in HRT arm. The third group included patients with a risk of >25% and the prostate together with the proximal 2 cm of the SV were treated to the maximum dose unless the SV were involved where in that case the maximum dose was delivered to the whole SV. Organs at risk included the femoral heads, penile bulb, bladder, bowel bag and rectum and mandatory dose constraints for these organs were defined. A planning target volume (PTV) of 1 cm to all directions and 5 mm posteriorly was used. The patients' position was evaluated and corrected by everyday KV imaging and cone beam CT once weekly. Patients in the first arm received 2.25 Gy/fr 5 days per week (Monday to Friday) and patients in the second arm received 2 Gy/fr 5 days per week. Biologically equivalent doses were calculated assuming an α/β ratio of 1.5 (BED=180 Gy in HRT and 172.7 Gy in CRT). All patients received treatment with VMAT technique. Randomization was performed by a random number generator using a web-based application.

### Outcomes

Acute toxicity was defined as an event that manifested during RT or within the first 3 months after the end of treatment. RTOG toxicity grading scale was used to evaluate the adverse events for genitourinary and gastrointestinal system. Toxicity was evaluated every week during RT and on weeks 11, 15 and 19 from start of RT. Quality of life was evaluated by patient reported questionnaires 18. Before start of RT, baseline scores were collected for each patient and a mean baseline value was calculated for the whole cohort. Patients were evaluated again on weeks 4, 11 and 19 from start of RT and the change in quality of life for each patient was determined by subtracting the new score from the baseline value.

### Statistical analysis

The toxicity evaluation results from RTOG forms were grouped into two categories, grade <2 and grade ≥ 2 for both GU and GI and we used Kaplan Meier curves to present them. Log rank test was used to compare the curves and Cox regression to identify any possible effect of baseline patient and treatment characteristics to toxicity. The variables included in the model were age, seminal vesicles invasion group, T stage, Gleason score, PSA, percentage of positive biopsy cores, hormone therapy, risk group. The mean values from the self-assessment questionnaires for each group were compared by T test or non-parametric Man Whitney U test. Statistical significance for all tests was set at 0.05. Descriptive statistics were used to analyze patient and treatment characteristics and SPSS version 25 for the statistical analysis.

## Results

**Figure 1:**
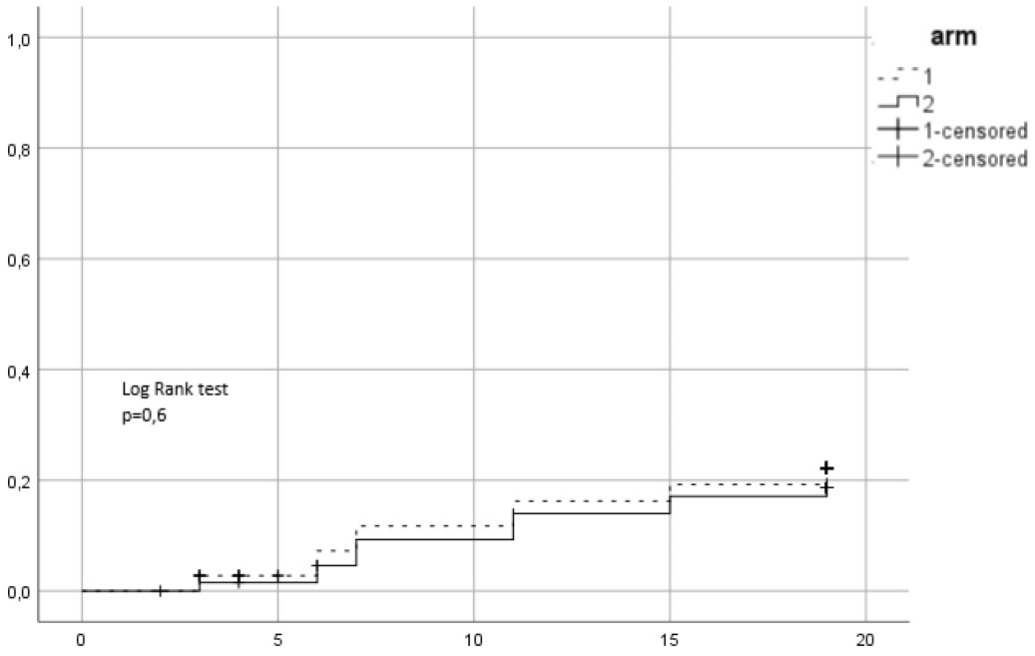
Cumulative incidence of acute grade ≥ 2 GU toxicity. Time in weeks from start of RT.

**Figure 2:**
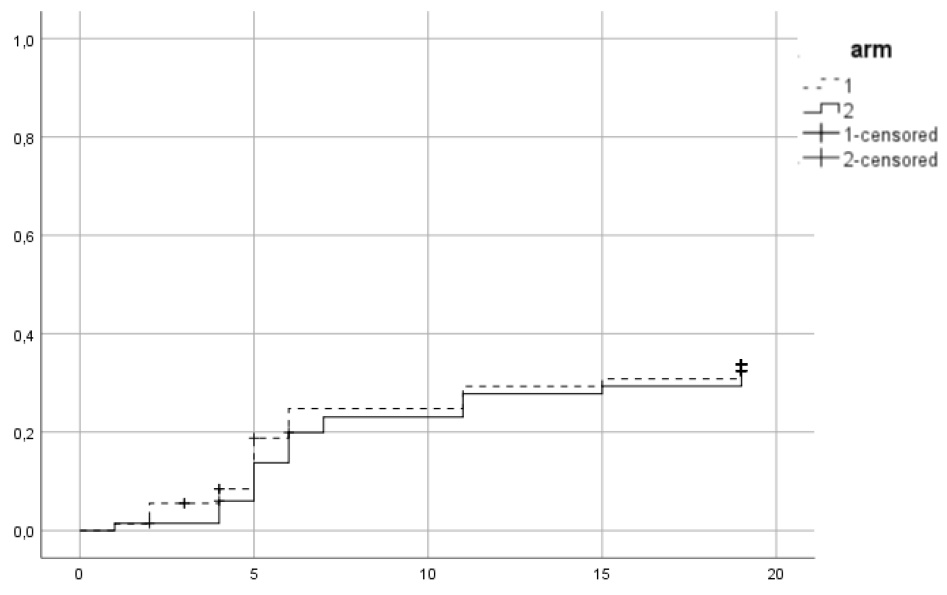
Cumulative incidence of acute grade ≥ 2 GI toxicity. Time in weeks from start of RT.

Between 2015 and 2016, 139 patients were recruited in the trial. 72 were assigned to the first arm and received hypofractionated RT (HRT) and 67 were assigned to the second arm of conventional RT (CRT). In CRT arm 64 completed allocated treatment, 1 patient decided to quit treatment and 2 withdrew due to other health reasons. In the HRT, 67 patients completed treatment, 1 quite due to urinary tract infection, 2 quit for unknown reasons and 2 were unsuitable to continue in the study due to the need of permanent urinary catheter. Patient characteristics are summarized in Table 1. 38 (27.3%) patients were low risk, 52 (37.4%) intermediate and 49 (35.3%) high risk. Mean PSA was 11.9 ng/ml and mean age 70.35 years. 96 (69.1%) patients received ADT. 86 patients (61.9%) belonged to SV group 1, 44 (31.7%) to group 2 and 9 (6.5%) to group 3. Acute urogenital toxicity grade ≥ 2 was observed in total in 44 patients from which 23 (31.9%) belonged to the HRT arm and 21 (31.3%) in CRT (Figure 1). There was no statistically significant difference between arms (p=0.79). For acute GI grade ≥2, the corresponding values were 15 (20.8%) and 12 (17.9%) (Figure 2) and no significant difference was identified either (p=0.6).

**Figure 3:**
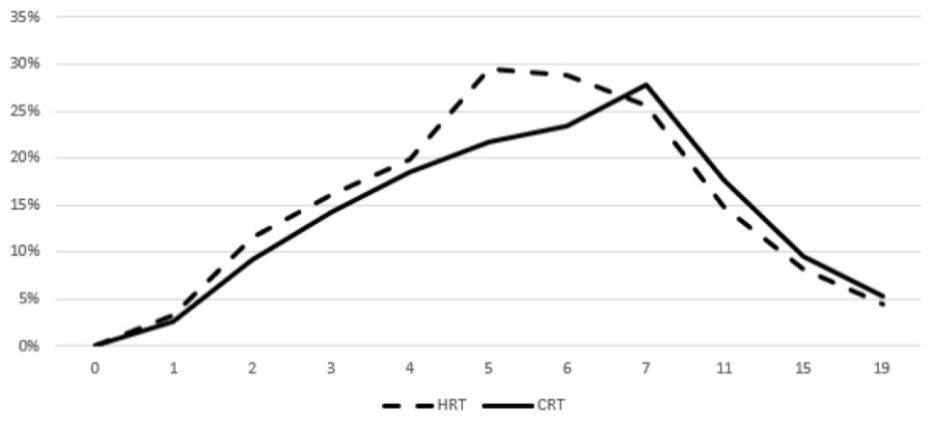
Prevalence of acute grade ≥ 2 GU toxicity. Time in weeks from start of RT.

**Figure 4:**
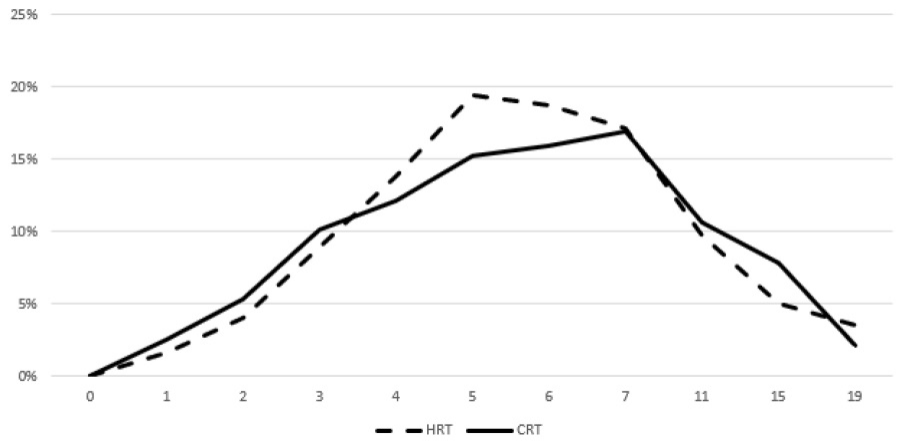
Prevalence of acute grade ≥ 2 GI toxicity. Time in weeks from start of RT.

Figures 3 and 4 show the prevalence of grade ≥ 2 GU and GI toxicity respectively. A gradual escalation of toxicity incidence is observed up to weeks 5 to 7 which represents the completion of treatment. Toxicity in the hypofractionated arm peaked on week 5 for both GI and GU and on week 7 for conventional fractionation. At the peak there was no statistical difference between arms (p=0.64 for GI and p=0.91 for GU). By week 19, patients in both arms had recovered almost completely, with percentages returning to the values observed during the first week of treatment.

**Figure 5:**
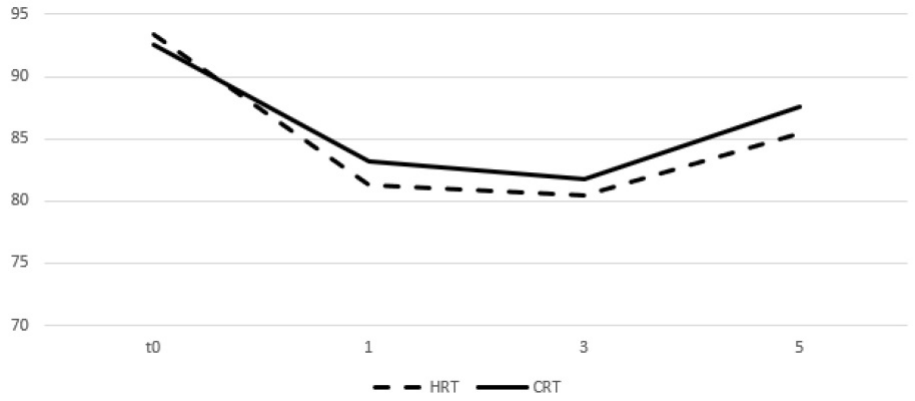
Change of means in relation to time for GU quality of life assessment. Time in months, t0 represents group baseline values before start of treatment.

**Figure 6:**
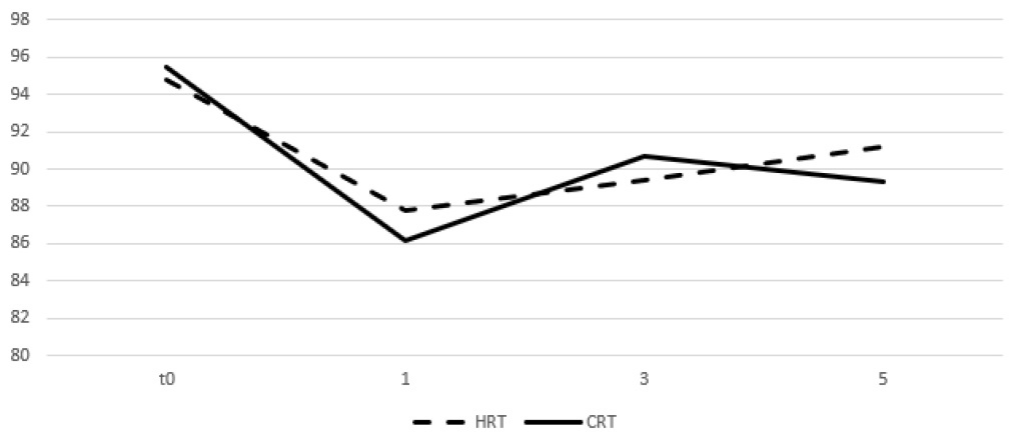
Change of means in relation to time for GI quality of life assessment. Time in months, t0 represents group baseline values before start of treatment.

Table 4 and figures 5 and 6 show the mean values of the scores for each group and time point derived from the self-assessment questionnaires for quality of life evaluation. A drop in the mean score was observed during the first month from start of RT for both arms and an increase was seen on months 3 and 5 with the values remaining relatively stable. After comparing the difference from the baseline score no significant differences were observed between groups for all the three time points.

## Discussion

In this trial we found that hypofractionated radiotherapy for prostate cancer with 2.25 Gy/fr to a total of 72 Gy is safe in terms of acute toxicity and well tolerated by the patients. There was no difference in acute toxicity from either GI or GU according to RTOG scale. Patients' Self-assessment questionnaires for toxic effect showed a decrease in the score during the first month of treatment for both arms, an observation which is reasonable since the first month represents the more acute phase of therapy and toxicity is expected to be higher during that period. During months 3 and 5 patients toxicity scores had substantially improved and we noticed no statistically significant differences between arms. The prevalence of grade ≥ 2 toxicity during treatment and for the first 3 months after completion showed an earlier, but not statistically significant, peak for hypofractionation and patients form both arms had recovered almost completely by 3 months. No association was observed between patient and treatment factors with toxicity. Interestingly, we observed no correlation between SV groups and GI toxicity, an observation seen in previous studies 19, 20. This could probably be explained by the lower biologically equivalent doses used in our study. Our schedule appears to be safer since toxicity was not influenced by difference in field size between SV groups, although this observation should be carefully interpreted since the size of our cohort is small and there are few patients in group 3.

Some small randomized trials have investigated the toxicity profile of hypofractionation in the management of prostate cancer. Pollack et. al. 21 found that a treatment plan of conventional 76 Gy is equal to 70.2 Gy in 2.7 Gy/fr in terms of biochemical control and toxicity but stated that men with compromised urinary function before treatment may not be ideal candidates for this approach. Toxicity was not statistically different in the study of Arcangeli et. al. 22 who compared 62 Gy in 20 fractions to 80 Gy in 40 fractions, with only a slight non-significant increase in tolerable and temporary acute toxicity. Similar results were observed in a comparison study of 75,6 Gy in 42 fractions with 72 Gy in 30 for acute toxicity, although late grade 2 and 3 events were significantly increased with hypofractionation.^23^ A more aggressive hypofractionation approach 24 of 3.15 Gy/fr to a total of 63 Gy was still not associated with worse toxicity outcomes, with GI and GU acute toxicity developing earlier and recovering faster using hypofractionation. There was an association between acute toxicity and bowel and urinary quality of life outcomes, but the authors concluded that longer follow up is needed to determine a possible significance of these associations with late toxicity.

Larger randomized trials treating patients with hypofractionation will help clarify this issue. The HYPRO trial 25 in Netherlands is a phase 3 trial which randomized 820 patients to either one of the following groups: conventional fractionation of 78 Gy (5 fractions per week) or hypofractionation of 64.6 Gy with 3.4 Gy/fr 3 fractions per week. One of the primary endpoints was to detect a non-inferiority of hypofractionation in cumulative incidence of grade 2 or worse acute and late genitourinary and gastrointestinal toxicity. Preliminary results were published after a median follow up of 60 months, and non-inferiority could not be confirmed. Specifically, the incidence of grade 2 or worse genitourinary toxicity at 3 years was 39% for the standard fractionation arm and 41.3% for the hypofractionation, with a HR of 1,16 (less than 1,1 was set by the authors as a cut off to reject inferiority). For grade 2 or worse gastrointestinal toxicity at 3 years the corresponding values were 17.7% vs 21.9% with a HR of 1.19. It is important to state though that more high-risk patients were included in this study compared to others, leading to more patients receiving prescribed dose to the whole seminal vesicles. Additionally, the high baseline rate of grade ≥ 2 GU is believed to have affected the high acute toxicity results observed. CHHIP trial 26 is another phase 3 non inferiority UK trial which recruited 3216 patients with localized T1-T3aN0M0 prostate cancer and were randomly assigned to conventional 74 Gy radiation therapy or one of two hypofractionated schedules (60 Gy in 20 fractions or 57 Gy in 19 fractions). The results on acute toxicity showed no significant difference in either proportion of cumulative incidence of side effects. However acute toxicity from both GI and GU peaked sooner in the hypofractionated arms than in the control (4-5-week vs 7-8 week) and during that peak there was significantly more acute bowel toxicity in hypofractionated schedules. RTOG 0415 is a large phase III trial comparing 73.8 Gy (1.8Gy/fr) of conventional fractionation to 70 Gy (2.5Gy/fr). After a median follow up of 5.8 years, results were published in 2016 showing that patients receiving HRT developed significantly more late GU and GI grade ≥2 toxicity 27. On the contrary, toxicity rates between the two RT schedules were similar in PROFIT trial which recruited 1206 patients (608 pts in 60 Gy (3Gy/fr) HRT arm vs 598 pts in 78 Gy (2Gy/fr) CRT arm) 28. After a median follow up of 6 years acute and late GI toxicity grade ≥2 for HRT and CRT was 16.7% vs 10.5% and 8.9% vs 13.7% respectively. The corresponding values for GU were 30.9% vs 31% and 22% vs 21.8%.

Unlike other cancer types, prostate cancer is considered to have an α/β ratio of 1.2 - 3 much lower than the commonly assumed value of 10 for other diseases. A recent meta-analysis of altered fractionation randomized studies up to 2017 confirmed this allegation 29. Specifically, the authors found values of 1.2 and 2.7 when assuming no effect of overall time treatment or assuming a loss of 0.31 Gy/day respectively. The low α/β value offers a potential benefit for hypofractionation, since prostate cancer appears to be more sensitive to fraction changes compared to normal tissues. A rise in the dose per fraction would cause a bigger increase in the biologically equivalent dose delivered to the prostate than that delivered to normal tissues. This is particularly important since dose escalation has been proven to improve disease free survival. Recently published results of RTOG study further support this statement 30. RTOG sought to determine whether dose escalation would improve disease control and overall survival 30. 79.2 Gy were compared to 70.2 Gy and after a median follow up of 8.4 years a benefit in biochemical and distant metastases free survival was observed in favor of 79.2 Gy without an improvement in overall survival. Our schedule of 32 fractions is well tolerated by the patients with limited toxicity from both GU and GI and delivers a biologically equivalent dose of 81 Gy in 1.8 Gy/fr. This is 13 fractions less compared to 45 daily treatments required with conventional 1.8 Gy fractionation. Such a reduction in treatment duration without increase of toxicity would be more convenient for the patient and would decongest the health care system by reducing the use of the services.

The limitations of our study are its relatively small size and the short follow up period. Additionally, this trial included favorable patients in terms of performance status and urinary function, so the results should not be extrapolated to patients with important comorbidities or impaired urinary function.

Here we present the preliminary results of our study and longer follow up is needed to assess late toxicity. However, the preliminary results seem to agree with most of the published studies based on longer follow up. Specifically concerning the toxicity profile, there have been studies which identified a correlation between acute and late toxicity 1, 25, 31, 32. This is important especially for prostate cancer since it is a slow growing disease with patients most commonly having a favorable prognosis, so late toxicity evaluation needs a longer follow up period. An important review identified all published reports of prostate cancer patients undergoing radiation therapy where acute and late toxicity was described 33. Interestingly they found that there was a statistically significant correlation of acute and late toxicity especially in reports with a large number of patients and long follow up. Some of those reports even described a possible mechanism for this association like an inflammation and injury which leads to fibrosis and finally to the development of late toxicity. The authors concluded that acute toxicity could be used as a surrogate to predict late toxicity in the future and this could help physicians identify patients who would benefit from a personalized care since they are at a higher risk of late toxicity manifestation. In our study acute toxicity wasn't different between arms which is probably foretelling of a non-statistical difference in late toxicity as well, an observation consistent with most of the studies on hypofractionation.

## Figures and Tables

**Table 1:** Patient and treatment factors at baseline

Characteristics	Total n=139 (%)	Conventional fractionation n=67 (%)	Hypofractionation n=72 (%)
**Age (mean)**	70.3	70.9	69.8
**Age categorical**			
<70 years	57(41)	27(40.3)	30(41.7)
≥ 70 years	82(59)	40(59.7)	42(58.3)
**Clinical stage**			
T1	60(43.2)	28(41.8)	32(44.4)
T2	70(50.4)	36(53.7)	34(47.2)
T3	9(6.5)	3(4.5)	6(8.3)
**Gleason Score**			
<6	60(43.2)	29(43.3)	31(43.1)
7	61(43.9)	31(46.3)	30(41.7)
8-9	18(12.9)	7(10.4)	11(15.3)
**PSA**			
<10 ng/ml	84(60.4)	39(58.2)	45(62.5)
10-20 ng/ml	32(23)	17(25.4)	15(20.8)
>20 ng/ml	23(16.5)	11(16.4)	12(16.7)
**>50% cores positive**			
Yes	55(39.6)	31(53.4)	24(34.8)
No	72(51.8)	27(46.6)	45(65.2)
**Hormone therapy**			
Yes	96(69.1)	47(73.4)	49(72.1)
No	36(25.9)	17(26.6)	19(27.9)
**Risk group**			
Low	38(27.3)	18(26.9)	20(27.8)
Intermediate	52(37.4)	28(41.8)	24(33.3)
High	49(35.3)	21(31.3)	28(38.9)
**SV involvement probability**			
<10%	86(61.9)	44(65.7)	42(58.3)
10-25%	44(31.7)	20(29.9)	24(33.3)
>25%	9(6.5)	3(4.5)	6(8.3)
**Type of treatment**			
Conventional	67(48.2)	-	-
hypofractionation	72(51.8)	-	-

**Table 2:** Univariate and multivariate Cox regression analysis for the association of patient and treatment factors with grade ≥ 2 acute GI toxicity

	Univariate		Multivariate	
Variables	p	OR(CI)	p	OR(CI)
**SV invasion risk group**				
1 vs 2	0.53	1.9(0.25-14.4)	-	-
1 vs 3	0.38	2.5(0.32-19.6)	-	-
Age (<70 vs ≥ 70)	0.76	0.89(0.42-1.9)	-	-
ADT (yes vs no)	0.54	0.13(0.56-0.29)	-	-
**T stage**				
T1 vs T2	0.3	0.28(0.38-21.5)	-	-
T1 vs T3	0.7	1.4(0.19-11.6)	-	-
**Risk group**				
Low vs intermediate	0.1	2.3(0.83-6.3)	-	-
Low vs high	0.26	1.8(0.65-4.8)	-	-
**PSA**				
<10 vs 10-20	0.69	1.3(0.38-4.5)	-	-
<10 vs >20	0.23	2.2(0.6-8.2)	-	-
**Gleason score**				
6 vs 7	0.23	2.5(0.6-10.9)	-	-
6 vs 8-9	0.56	1.6(0.35-7.1)	-	-
Positive biopsy cores (<50% vs ≥ 50%)	0.46	1.35(0.6-3.07)	-	-

SV: seminal vesicles, ADT: androgen deprivation therapy, PSA: prostate specific antigen

**Table 3:** Univariate and multivariate Cox regression analysis for the association of patient and treatment factors with grade ≥ 2 acute GU toxicity

	Univariate		Multivariate	
Variables	p	OR(CI)	p	OR(CI)
**SV invasion risk group**				
1 vs 2	0.21	0.54(0.21-1.41)	-	-
1 vs 3	0.19	0.5(0.17-1.42)	-	-
Age (<70 vs ≥ 70)	0.76	0.91(0.5-1.65)		
ADT (yes vs no)	0.17	0.52(0.2-1.33)	0.16	0.5(0.19-1.32)
**T stage**				
T1 vs T2	0.69	0.82(0.31-2.2)	0.51	0.67(0.2-2.2)
T1 vs T3	0.09	0.41(0.15-1.14)	0.09	0.36(0.11-1.18)
**Risk group**				
Low vs intermediate	0.59	1.2(0.59-2.5)	-	-
Low vs high	0.63	0.83(0.41-1.71)	-	-
PSA				
<10 vs 10-20	0.05	3.26(1-10.63)	0.01	8.32(1.62-42.75)
<10 vs >20	0.17	2.5(0.68-9.2)	0.04	5.18(1.05-25.61)
**Gleason score**				
6 vs 7	0.78	0.89(0.4-2)	0.15	0.46(0.16-1.32)
6 vs 8-9	0.1	0.48(0.19-1.16)	0.13	0.44(0.16-1.25)
Positive biopsy cores (<50% vs ≥ 50%	0.14	1.65(0.85-3.2)	0.015	3.08(1.23-7.66)

SV: seminal vesicles, ADT: androgen deprivation therapy, PSA: prostate specific antigen

**Table 4:** Mean values of quality of life assessment scores and comparison between treatment groups

	4^th^ week			11^th^ week			19^th^ week		
	HRT	CRT	p	HRT	CRT	p	HRT	CRT	p
Genitourinary	81.3	83.2	0.7	80.4	81.8	0.54	85.4	87.6	0.32
Gastrointestinal	87.8	86.2	0.052	89.4	90.7	0.42	91.2	89.3	0.98
